# BanglaNewsClassifier: A machine learning approach for news classification in Bangla Newspapers using hybrid stacking classifiers

**DOI:** 10.1371/journal.pone.0321291

**Published:** 2025-06-09

**Authors:** Tanzir Hossain, Ar-Rafi Islam, Md Humaion Kabir Mehedi, Annajiat Alim Rasel, M. Abdullah-AL-Wadud, Jia Uddin

**Affiliations:** 1 Department of Computer Science and Engineering, BRAC University, Dhaka, Bangladesh; 2 Department of Software Engineering, College of Computer and Information Sciences, King Saud University, Riyadh, Saudi Arabia; 3 AI and Big Data Department, Endicott College, Woosong University, Daejeon, Republic of Korea; Kitami Institute of Technology, JAPAN

## Abstract

Bangla news floods the web, and the need for smarter and more efficient classification techniques is greater than ever. Previous studies mostly focused on traditional models, overlooking the potential of hybrid techniques to handle the ever-growing complex dataset and its linguistic patterns in Bangla to achieve higher accuracy. Addressing the challenge, this study presents a comprehensive approach to classify Bangla news articles into eight distinct categories using various machine learning and deep learning techniques. The use of traditional machine learning algorithms, deep learning architectures, and hybrid models, including novel stacking classifiers, was a part of our experiment. This study utilized a dataset of 118,404 Bangla news articles, applying rigorous feature extraction techniques including TF-IDF vectorization and word2Vec embeddings. Our best-performing model, a stacking meta-classifier combining bidirectional long short-term memory and support vector machine, achieved a remarkable 94% accuracy, leaving all basic models’ performance behind. Also, we provided an in-depth analysis of model performances, including confusion matrices, ROC curves, and error analysis, offering insights into the strengths and limitations of each approach. This research contributes significantly to the field of Bangla natural language processing and demonstrates the efficacy of ensemble methods and deep learning in news classification for low-resource languages.

## 1 Introduction

News topic classification with widespread applications has become a highlighted task in the quickly evolving field of natural language processing (NLP). This task of news classification involves the categorization of news articles into specific genres like politics, entertainment, sports, business, and many more. Over time this task has played an important role in driving the evolution of various machine learning (ML) algorithms. It has influenced the development of topic modeling techniques [1] and contributed to advancements in deep learning (DL) models [2,3]. Furthermore, the classification of news topics serves as an important downstream task for evaluating the performance of large language models (LLMs) in both fine-tuning and prompt-based paradigms [4,5]. Despite the popularity of the task in benchmarking language models (LMs), most of the evaluation has focused on English and a few other high-resource languages. As a result, it is uncertain how this approach extends to pre-trained multilingual language models for low-resource languages like Bangla. For instance, mBERT [6] and XLM-R [7] were pre-trained in multiple languages, including Bangla. However, an extensive evaluation of Bangla was not performed due to a lack of evaluation datasets. In general, only a handful of NLP tasks such as machine translation [8], named entity recognition [9], and sentiment classification [10] have standardized benchmark datasets for Bangla.

However, human-annotated datasets for benchmarking topic classification using language models for Bangla are scarce. Although some efforts have been made in this direction [1], there is still a significant gap in comprehensive standardized datasets for Bangla news topic classification. This results in negation when assessing the performance of multilingual models in low-resource languages like Bangla. Bangla, is the seventh most spoken language in the world, the mother language of Bangladesh, and one of the 22 scheduled languages of India. With distinct phonetic and syntactic features, the language Bangla is characterized. Also, it includes relatively free word order and a complex system of honorifics, nouns, verbs, and conjunctions. However, this language is grossly underrepresented in the field of NLP compared to languages such as English or Chinese. This disparity occurs due to the lack of high-quality annotated datasets. Even when datasets are available, they often lack quantity, and quality and are not up-to-date. The authors [11,12], have made significant contributions but faced several challenges. Firstly, limited dataset size and diversity; secondly, lack of comprehensive exploratory data analysis specific to Bangla; and thirdly, a narrow focus on a few machine learning models. Also, the absence of model interpretability, inadequate Bangla-specific preprocessing techniques, and limited use of advanced ensemble methods are also part of the challenges faced. Furthermore, many studies relied on manually annotated datasets, which can be time-consuming and prone to inconsistencies. The lack of standardized, large-scale Bangla datasets has hindered the development of robust NLP models capable of handling the language’s complexity. Additionally, most studies have not fully explored the potential of combining multiple models or we can say leveraging the unique linguistic features of Bangla in their preprocessing steps. As a result, these limitations have constrained the progress of Bangla NLP. Eventually, creating a significant gap to fill in areas such as text classification, sentiment analysis, and other language processing tasks.

We are living in a time which is characterized by a huge amount of online news sources. The ability to accurately categorize and analyze news content through news classification extends far beyond academic interests, playing an important role in real-world applications. The classification of news articles opens many ways for not only the professional, but also the daily consumers of the news. To explain, researchers, analysts, and media professionals can monitor how topics are being presented across different genres, collecting valuable information like trends, and public discourse. Also, a news article can be manipulated in many ways to influence readers; the ability to accurately classify and analyze the news content gives us the opportunity to foster media literacy and empower the readers [13]. For example, suppose that there is a news article titled “Government Launches Sports Initiative Amid Political Tensions" and through our classification model, we got a topic breakdown like Politics: 60%, Sports: 30%, and Economy: 10%. The headline might suggest the article to be sports-related, but the classification helps the readers to realize that the article predominantly focuses on the political context. This also suggests that the story uses the sports initiative to frame or highlight certain political narratives, swaying the reader’s perception of the government or the political situation. By developing robust classification models, especially for Bangla, we contribute to the cause and empower the readers to identify when a seemingly neutral, in this case, sports-related, article is actually heavily influenced by political content. Eventually, our work promotes media literacy by enabling readers to assess how much of the article is truly or how much it matches with what it indicates.

Our research methodology involved experimenting with fundamental models like naive bayes and logistic regression, as well as implementing sophisticated ensemble techniques. We found that a combination of traditional algorithms like support vector machine (SVM), naive bayes (NB), and logistic regression (LR) with advanced neural networks, including convolutional neural networks (CNN) and bidirectional long short-term memory (BiLSTM) models as base classifiers with a meta-classifier fine-tuned on their outputs, performed exceptionally well, capitalizing on the complementary strengths of various classification methods. This system achieves high accuracy in categorizing Bangla news articles.

To address these gaps, our research aims to make significant contributions to the field of Bangla news classification:

- **Dataset contribution:** We introduced a high-quality dataset consisting of 118,404 Bangla news with eight distinct categories which is one of the largest Bangla datasets in comparison with previous datasets. Also, the dataset uses pre-labeled articles from reputable Bangla news sources ensuring high labeling accuracy and diversity while saving annotation costs.
- **Comprehensive Exploratory Data Analysis:** A detailed analysis of the model performance is shown in our study. Not only did we use matrices like confusion matrices, and ROC curves but also provided error analysis with category-specific insights. Identifying specific challenges. On the other hand, very few studies emphasize the interpretability of Bangla NLP.
- **Model development and comparison:** We have trained and compared eight different ML models, four different stacking classifiers, and two soft voting classifiers to evaluate their performance differences.
- **Interpretable models:** We have developed models that can provide explanations for their classification decisions.
- **Bangla-specific preprocessing techniques:** In our study, we used preprocessing techniques focusing on the Bangla language such as stop-word removal, punctuation handling, stemming, and word embedding. While other studies applied generalized preprocessing pipelines without considering Bangla-specific complexities.
- **Advanced ensemble method:** An advanced ensemble method is introduced in our study. This hybrid stacking classifier combines the modeling power of BiLSTM with the high-dimensional space-handling capability of SVM. The combination reduces the weaknesses of standalone traditional machine learning models while significantly improving classification accuracy and robustness.

Furthermore, in Literature review section we tried to have a comprehensive literature review, examining relevant studies in news classification across various languages and highlighting the gap in Bangla news classification research. After that, Methodology section provides a detailed methodology, including data collection, preprocessing techniques, feature extraction methods, and the diverse range of classifiers employed in our study. In Results and discussion section we presented our results and offered an in-depth discussion, analyzing the performance of different models through various metrics, ROC curves, and error analysis. We also explore feature importance and the trade-offs between model complexity and performance. Finally, concluding the paper, by summarizing our key findings, discussing the implications of our work, and suggesting directions for future research in Bangla news classification and low-resource language NLP in Conclusion and future work section.

Through this comprehensive approach, we aim to Bangla news classification and contribute to a more inclusive and comprehensive understanding of NLP capabilities in diverse linguistic contexts. Our work not only advances the field of Bangla NLP but also has the potential to empower readers with better tools for media literacy and critical news consumption.

## 2 Literature review

The spread of biased and misleading information in news articles leads researchers to give their best efforts to solve such problems. We present some related works in this direction. The authors of the paper [14] have introduced MAD-TSC, the first large multilingual aligned dataset designed for target-dependent sentiment classification in news articles across eight languages. This dataset was created from a parallel news corpus with professional translations. Mad-TSC contains over 5100 annotated entities mentioning 4714 sentences, with rigorous consolidation of crowd-sourced sentiment annotations eventually ensuring high quality. If we compare the MAD-TSC with existing news TSC datasets, MAD-TSC covers a wider geographic distribution of sources, containing more complex examples with longer sentences, and less skewed mention distribution. The authors mentioned that they found MAD-TSC more challenging than prior datasets, with significant performance variability across languages. Moreover, they found that machine translations can effectively replace human translation efforts, enabling the creation of training data in other languages from an English dataset as well as translating test sets to English to leverage the best available English models during inference. To highlight the main contributions of the paper, the introduction of MAD-TSC, quantifying multilingual TSC performance, machine translation’s feasibility for extending English datasets, and comparing complexities with prior work.

Ahmed Qarqaz and Malak Abdullah in their research [15] tried to showcase a comprehensive exploration of transformer-based language models for news article classification. Through their study they contributed to the demonstration of the effectiveness of the “Longformer” transformer model. Moreover, it achieved a notable F1 micro score of 0.256 and an F1 macro score of 0.442 on SemEval-2023 Task 3, subtask-1, and subtask-2 respectively on the English dataset. The whole process took various state-of-the-art models and emphasized the importance of fine-grained analysis and preprocessing pipelines in increasing classification accuracy. Their findings emphasize the advantages of transfer learning and large pre-trained models for improved performance in downstream applications. Their findings underscore the importance of transfer learning and pre-trained models, however, their study lacks application to low-resource languages, including Bangla. JUSTR00 team has contributed significantly to the field of natural language processing through meticulous experimentation and insightful discussions. Eventually, setting a benchmark for future research endeavors in news article classification.

Alami *et al*. in the paper [16] have presented a comprehensive approach for English document genre classification in the context of SemEval-2023 Task3. In subtask 1 he utilized an ensemble technique combining Longformer, RoBERTa, GCN, and a sentence number-based model. Moreover, his study addressed the crucial task of detecting genre, framing, and persuasion techniques in online news articles, eventually contributing to the field of AI-based media analysis. The authors achieved a promising 12th rank in the document genre classification for English text by optimizing each model on simple and easy-to-understand objectives and got Longformer: F1 macro = 0.31932, F1 micro = 0.57407 RoBERTa: F1 macro = 0.31932, F1 micro = 0.57407 GCN: F1 macro = 0.28802, F1 micro = 0.61111 Sentence number-based model: F1 macro = 0.17198, F1 micro = 0.18519 Ensemble: F1 macro = 0.39351, F1 micro = 0.51852 scores. With the help of transformer-based models and a graph convolutional network, along with a novel sentence number-based model, the approach has shown the effectiveness of ensemble methods in improving classification accuracy and showcasing the potential of AI in media analytics. However, the study focused on high-resource languages and did not address low-resource settings like Bangla.

Along the same lines, the study [17] has explored the use of AdapterFusion layers to enhance multilingual news genre classification. The authors have utilized an adapter-based model within the BERT architecture to address the multilingual nature of the task, helping them to categorize news articles into reporting, opinion pieces, or satire in the SemEval-2023 Task 3. For each language, they trained a task-specific adapter and by using AdapterFusion to combine these adapters, eventually leveraged language-specific knowledge. This method clearly denotes significant improvements, with their model achieving a notable F1 macro score of 0.5942 for English, placing 4th out of 27 teams, and 0.7504 for Greek in zero-shot scenarios, securing 3rd place out of 15 teams. For some languages, it showed strong performance; however, less stability was also seen for some others. The main highlight of the study was the potential of AdapterFusion in improving multilingual NLP tasks. Suggesting further enhancements by incorporating more languages and optimizing the AdapterFusion setup.

POLITICS, XLM-RoBERTa, and Longformer have been used in this study [18] to approach the news genre categorization subtask of SemEval-2023 Task 3. As the challenge of the task is to classify news articles into three categories: opinion, reporting, and satire, across multiple languages the authors have utilized POLITICS. It is a pre-trained language model trained on 3.6 million English political news articles. They fine-tuned POLITICS for this specific task. Also, they addressed the multilingual challenge by translating all articles into English before processing. Their system performed very well and ranked first on the English dataset with an F1 macro score of 78.43, significantly outperforming the second-best system by 16.8 points. For other languages, the performance was less consistent and got 6th place, which indicates that there are areas of improvement in multilingual capabilities. The superiority of domain-specific, large-scale pre-trained models for such tasks was the main highlight of the study. It also emphasized the necessity for enhanced multilingual models and datasets. Moreover, sliding windows for long document coverage and mean pooling for aggregating information were a part of their method. They also tested alternative models like XLM-RoBERTa and Longformer, although POLITICS remained the top performer. Effective techniques for low-resource multilingual text classification and providing ways of handling genre categorization in news articles were the main goals of the paper.

Additionally, Mittal *et al*. in their study [19] introduced an extensive Marathi text classification corpus across 12 categories and varying text lengths (short, medium, long). Moreover, the authors try to evaluate the state-of-the-art BERT models, with MahaBERT outperforming others. The work not only addresses the lack of such datasets in the low-resource Marathi language but also provides insights into model performance. However, it lacks details on data preprocessing and hyperparameter tuning. To explore other domains, architectures, transfer learning techniques, and interpretability aspects could be considered as opportunities. This paper’s findings on the impact of text length and the comparison with BERT-based models helped in our Bangla newspaper classification work. While their work highlights the importance of language-specific models and preprocessing techniques. Also, their focus on BERT-based models makes it computationally demanding, which in the case of low-resource contexts like Bangla may not be feasible. Also, the gaps in the paper identified that leveraging multi-task learning or manual dataset verification can help the hybrid model’s performance in the low-resource Bangla setting.

FastFit was introduced in this paper [20]. It was a novel method for fast and accurate few-shot text classification tasks involving many classes. FastFit provides an upper hand in fine-tuning methods in terms of training speed and throughput in comparison with large language models like SetFit, Transformers, Flan, LLaMA, and Mistral. FastFit have shown significant improvements in classification performance by leveraging small language models and optimizing training processes. Eventually turning into a promising text classification tool. On one hand, FastFit excels in speed and accuracy but on the other hand, there are still opportunities to explore like, its application in speech recognition domains.

Summarizing the paper [21] we got to know that it focuses on classifying fake news in Malayalam using a random forest ML model. Many studies have explored various machine learning algorithms for fake news detection using naive bayes, LSTM, and support vector machines, eventually showing the importance of accurate classification methods. However, using ensemble models like random forest aligns with existing research and shows its effectiveness in identifying false information. Also, the study emphasizes the importance of language-specific models in addressing misinformation, especially for diverse linguistic contexts, showing the need for tailored approaches in different language settings.

The paper [22] has introduced a comprehensive study on fake news detection in the Malayalam language. They have explored machine learning, deep learning, and transformer-based models. Tanzim Rahman *et al*. in their paper has critically analyzed the effectiveness of various approaches, among them the Malayalam BERT model achieved the highest macro F1 score of 0.88 and provides insights into the challenges and limitations of pre-trained models in low-resource scenarios. On the one hand, the paper has emphasized the importance of language-specific models on the other hand they conducted a thorough error analysis. They also mentioned future work opportunities through exploring ensemble models, improving interpretability, and domain-specific fine-tuning, specifically in multilingual settings.

The paper [24] conducted a comprehensive examination of a comprehensive approach to classify Swahili news articles. They used machine learning models and explainable AI techniques. They combined the newly scraped web articles with the existing data to create a large dataset and evaluated the performance of classical machine learning models, ensemble models, and deep neural networks like CNN and LSTM variants. Most of the models got an accuracy of over 75%. Furthermore, the authors tried to provide explanations for the model’s prediction through Local Interpretable Model-Agnostic Explanations or LIME. With the use of explainable AI techniques the ‘black box’ nature of complex models cake out and it is a significant contribution to low-resource Swahili language.

The paper by [25] Hussain *et al*. has tried to present a comparative analysis using support vector machine (SVM) and logistic regression (LR) classifiers for categorizing Bangla (Bengali) news articles. Around 12,500 Bangla news articles were used to collect datasets from various online sources, covering 20 categories. An accuracy of 84% was achieved through SVM with a linear kernel, outperforming LR with 81% accuracy on the top 12 news categories. Moreover, the paper shows a comprehensive review of related work on text classification for low-resource languages like Bangla. It also highlights the use of techniques such as decision trees, naive bayes, neural networks, and transformer models. While their study highlighted the effectiveness of classical machine learning methods. However, it did not explore hybrid models or leverage larger datasets. Thus leaving room for improvement in accuracy and generalizability with ensemble methods.

Hashmi *et al*. in their study [23] introduced a comprehensive approach for advancing fake news detection through the integration of machine learning. They have also used deep learning and transformer-based models, eventually utilizing FastText word embedding and explainable AI techniques. The study addresses the challenges of misinformation spread on social media and also contributes to the field with increasing accuracy. Systematic data processing and model optimization on three benchmark datasets were mentioned in the paper’s methodology. The authors also highlighted the importance of interpretability and transparency in model decision-making from previous studies. Overall, this work demonstrated the benefits of combining CNN’s feature extraction capabilities with LSTM’s sequence modeling however, it focused only on binary classification tasks, eventually limiting its direct applicability to multi-class Bangla news classification. The summary of the related work can be found in Table 1.

**Table 1:** Summary of state-of-the-art models.

Algorithms	Dataset	Performance	Limitation
Roberta, Bert variants [14]	MAD-TSC	TD (Target Dependent) method on the English subset of MAD-TSC, with a macro F1 score of 73.2%.	Lacks evaluation on non-English and low-resource languages. Heavy reliance on machine translations.
English Dataset: bert-base-cased, roberta-base, bigbird-base, Longformer Multi-lingual Models: mbert-base-cased, XLM-roberta-base [15]	multi-lingual dataset annotated into three schemes for each subtask at SemEval-2023	Italian with an F1-Micro Score of 0.779.	Variability in multilingual performance and high computational costs of pre-trained transformers.
Longformer RoBERTa Graph Convolutional Network (GCN) Sentence number-based model [16]	SemEval-2023 Task 3 for news genre classification	Best F1-Macro: Ensemble (0.39351) Best F1-Micro: GCN (0.61111)	Ensembles with low F1-Macro, inconsistent model performance.
Longformer RoBERTa Graph Convolutional Network (GCN) Sentence number-based model[17]	SemEval-2023 Task 3 for news genre classification	The ensemble model achieved an F1 macro score of 39.35%	Limited evaluation across languages and genres and suboptimal ensemble F1 Macro.
POLITICS, XLM-RoBERTa, Longformer [18]	The dataset used includes articles in six languages (English, French, German, Italian, Polish, and Russian) for training, and three surprise languages (Spanish, Greek, and Georgian) for evaluation	For known languages: Longformer-4096 (69.96) For surprise languages: POLITICS-512 (66.04)	Translation artifacts reduce data quality and poor handling of surprise languages.
MahaBERT, IndicBERT, and MuRIL [19]	L3Cube-MahaNews	MahaBERT with 94.780%	Lacks hyperparameter tuning with limited generalization as the only focus was on the Marathi language.
FastFit-Small, FastFit-Large, Classifier-B, mT5-B T2T, mT5-B Enc [20]	Clinc150, Banking77, Hwu64, MASSIVE	FastFit-Large with an average of 88.2%	FastFit is not tested on diverse datasets. Also, limited adaptability to non-text domains.
Random Forest [21]	Shared_task (DravidianLangTech@EACL 2024)	The accuracy achieved by their model was 0.71 macro F1	Relies on a single Random Forest model and limited multilingual applicability.
Logistic Regression (LR), Decision Tree (DT), Naïve Bayes (NB), Convolutional Neural Network (CNN), Bidirectional Long Short-Term Memory (BiLSTM), m-BERT, and Malayalam-BERT [22]	Malayalam fake news dataset	Malayalam-BERT with 0.88	Limited exploration of hybrid ensemble methods. The high computational cost of Malayalam-BERT.
CNN-LSTM hybrid model combined with FastText embeddings [23]	WELFake, FakeNewsNet, FakeNewsPrediction	Achieved an accuracy of 0.99	Lacks diverse dataset validation, and low interpretability of deep models.
SVM, Logistic Regression, Multinomial Naive Bayes, Random Forest, Gradient Boosting, Hard Voting, Bagging, CNN-BiLSTM + Attention [24]	Swahili news classification dataset	CNN-BiLSTM + Attention model with a 97% AUC score	Significant class imbalance. Lack of advanced transformer models.
Support Vector Machine (SVM) and Logistic Regression (LR) [25]	The dataset comprises 11,570 Bangla news articles distributed across 12 distinct categories.	SVM with 84%	Lacks exploration of modern deep learning techniques.

Though there are several notable advancements in news classification across various languages, research specifically targeting Bangla news classification remains relatively scarce. To be more precise, previous studies have predominantly relied on either traditional machine learning techniques or standalone deep learning approaches, resulting in low performance and overlooking the potential of ensemble methods. In addition, many of these studies lacked large-scale annotated datasets and eventually failed to incorporate Bangla-specific linguistic features during preprocessing. Furthermore, model interpretability, a critical aspect of NLP, has also been largely underexplored in the context of Bangla and has resulted in limiting the ability to explain classification outcomes effectively. Some research has shown promising results using transformer-based architectures, however, their application to low-resource languages like Bangla is still in the early stages. Furthermore, there remains a noticeable absence of comparative studies evaluating hybrid models for Bangla news classification. Our study aims to bridge these gaps by proposing a hybrid stacking classifier that combines traditional and deep learning models to improve classification accuracy. With the introduction of a comprehensive dataset, the conduct of a detailed error analysis and providing insight into the interpretability of the model, our research significantly advances the field of Bangla NLP and its applications in news classification.

## 3 Methodology

**Fig 1:**
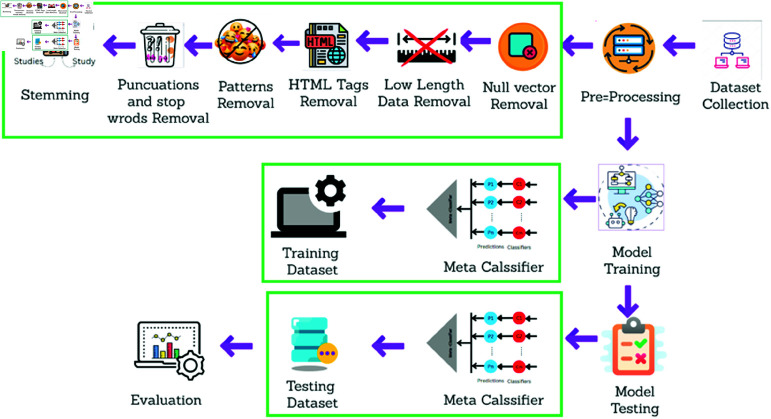
Proposed Framework of this research includes data collection, pre-processing, model training, and testing.

The target of news classification is to allocate news articles to their distinct categories as per the content. The proposed methodology followed in this research comprises data acquisition and pre-processing before the model training begins, along with analysis at the end. The overall process that took place in this experiment is shown in Fig 1.

### 3.1 Data collection

For this research, we scraped Bangla news articles from three major websites: Kaler Kantho, Daily Naya Diganta, and Jugantor. The data collection process was conducted in full compliance with the terms and conditions of these websites. Also, we thoroughly reviewed the terms and conditions of these websites to ensure that the use of their content for academic research purposes aligns with their policies. Also, were done strictly for non-commercial, academic purposes, adhering to ethical research practices and managing transparency throughout the process. Each website was scraped separately using Selenium, with custom scripts tailored to their unique structures. To ensure smooth scraping and avoid server bans, we implemented intervals between requests. The scraping process covered a span of approximately 8-9 days, capturing a total of 118,404 articles at a basic interval of 1-3 seconds per interval.

**Fig 2:**
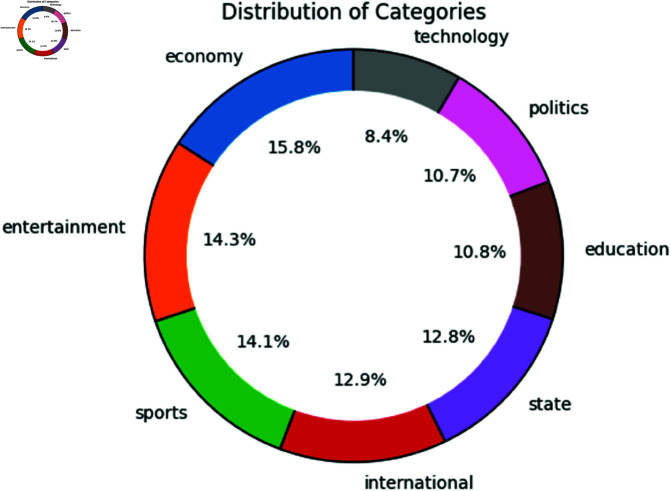
Dataset categories distribution.

The dataset is divided into 8 classes such as political news, technology news, educational news, state news, international news, sports news, entertainment news, and lastly economic news shown in Fig 2. The distribution of data is as follows, Economic news: 15.8%, Technology news: 8.4%, Political news: 10.7%, Educational news: 10.8%, State news: 12.8%, International news: 12.9%, Sports news: 14.1%, Entertainment news: 14.3%.

While the dataset is relatively balanced, the 7.4% difference between the highest category (Economic news, 15.8%) and the lowest category (Technology news, 8.4%) indicates a mild imbalance. In a large-scale dataset like ours where each category has sufficient data we determined any extra steps unnecessary to address class imbalance. Also, The dataset utilized pre-existing labels from the source news outlets which ensured reliable categorization without the subjectivity of manual annotation Because these news outlets harbors dedicated journalists thus reducing the human induced error in the preprocessing step. In contrast, despite having quality annotation The dataset is derived from a limited number of news outlets. A big chunk of articles are written by the same journalists, thus sources may reflect specific ideological, political, or cultural biases. Some articles span over multiple themes but the sources forced them into a single category. This limits our dataset ability for multilabel classification.

### 3.2 Data pre-processing

Firstly, we have collected various Bangla News articles from various online news sources using the Selenium web scraping library in Python. Collected data was then saved in CSV format using the pandas library in Python. The CSV file contained web-scraper-start-url, Links-href, category, and lastly the text description of the Bangla news articles. The reason behind this was that we wanted to classify our text data so we dropped the web-scraper-start-url, Links-href. Then using a label encoder from the scit learn library we transformed categorical data into numerical values. For instance, “sports news" = 1 “technology news" = 2 “economic news" = 3.

**Fig 3:**
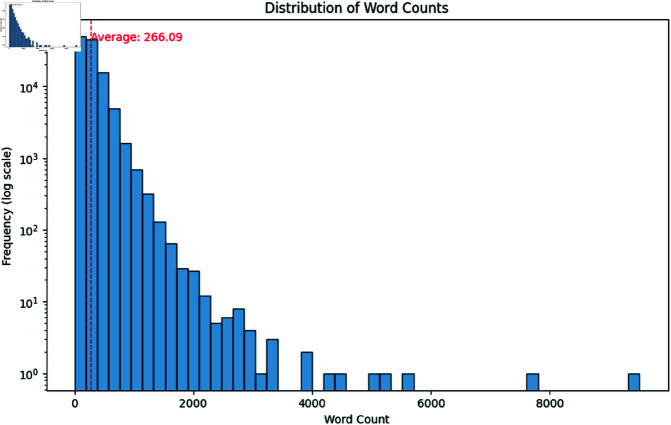
Distribution of (a) word counts and (b) character length.

Moreover, doing further investigation we found that the text data contains some HTML tags which we removed using the regular expression library. After removing the HTML tags we dropped the column containing less than 50 characters because we deemed it unnecessary for our model training as our dataset contains on average 1767.52 characters shown in Fig 3. After seeing the character distribution of news articles, the choice of 50 characters as the threshold represents a balance between retaining enough characters to provide adequate context while filtering out short texts that may not contribute significantly to the model’s learning process.

Also, we used regular expressions to match and replace Unicode characters representing emoticons, symbols, pictographs, transport and map symbols, flags, and some punctuation marks. Also using the same process we removed the punctuation marks. The reason behind this is that we found that punctuation marks do not carry much semantic meaning for text classification tasks, from previous text classification papers. They often introduce noise or unwanted variations that are not very useful for the classification model.

**Fig 4:**
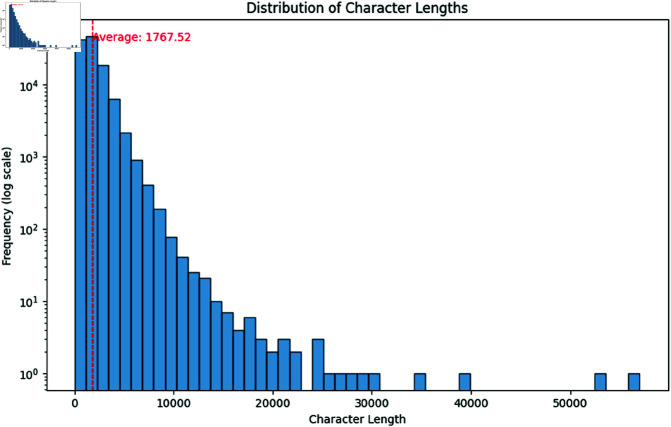
Word cloud before pre-processing.

**Fig 5:**
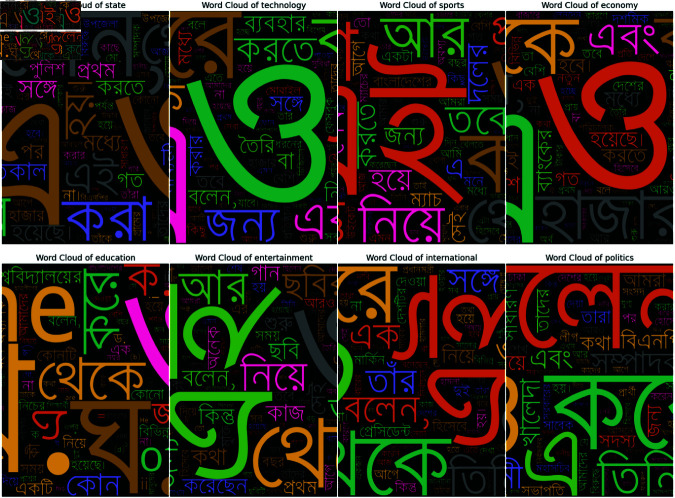
Word cloud after pre-processing.

After completing all of these pre-processing steps we found that some of our data still contained some noises like “ (backslash)” so we removed those from the datasets. Furthermore, we removed stop words from our dataset. By removing stop words, the remaining words in the text tend to be more meaningful and distinctive for the classification task so it helps the model to focus on important words. For removing stop words, we utilized a curated list of 800 Bangla stop words sourced from a GitHub repository Bangla Stopwords Dataset. Then the matching stop words were removed using regular expressions from our dataset. Also, we used bangla-stemmer library to reduce the dimensionality of the feature space of our dataset. After reviewing the documentation of Bangla Stemmer Library, we found that it uses a rule-based approach to reduce words to their root forms. The rules file in this library is structured in blocks, with each block enclosed within curly braces ({}). These blocks contain multiple rules, arranged by their priority, and the stemming process involves applying these rules sequentially over multiple passes. 1. Structure of the Rules, 2. Single-character removal, 3. Character replacement, 4. Complex replacements. In case a word matches multiple rules, the first matching rule is applied by default. After doing all those processes, our dataset was reduced from 118917 to 118404 rows. Also, Tokenization in this project was performed by splitting text into individual words using the .split() method, providing a basic approach to word-level tokenization. Because we have already handled punctuation, mixed-script text, or other complexities like emojis this simple method works well. Then to further capture contextual patterns, we used NLTK’s (Python Library) ngrams function to generate sequences of n consecutive tokens. After that, we constructed two, word clouds to compare the dataset before pre-processing in Fig 4 and after pre-processing in Fig 5.

Furthermore, for a better understanding of the dataset and the distribution of the content lengths we analyzed the word, character, and text counts across all articles. The histograms provided below illustrate these distributions and highlight the skewness and the presence of outliers in the dataset. It also helps to visualize the methodology providing a better understanding of the data characteristics.

### 3.3 Features extraction

**Fig 6:**
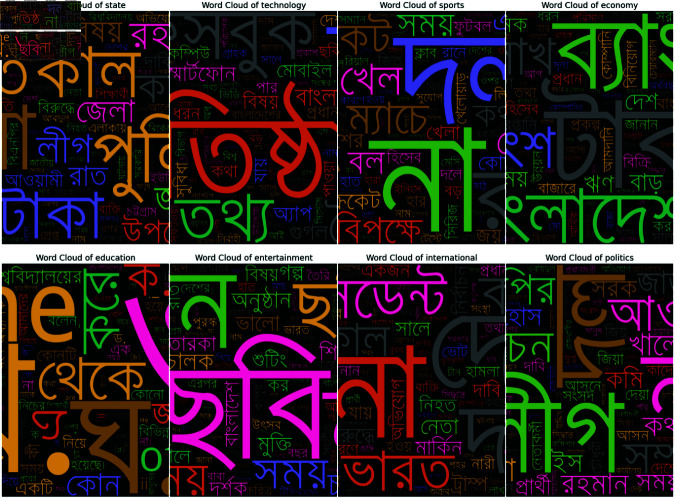
Global feature extraction process.

Our BanglaNewsClassifier employs a multi-faceted approach to feature extraction. It combines traditional methods with deep learning techniques to extract the features. This section details all the various feature extractors we employed to enable effective classification across multiple categories. The whole feature extraction process is shown in the Fig 6

#### 3.3.1 Lexical features.

**Fig 7:**
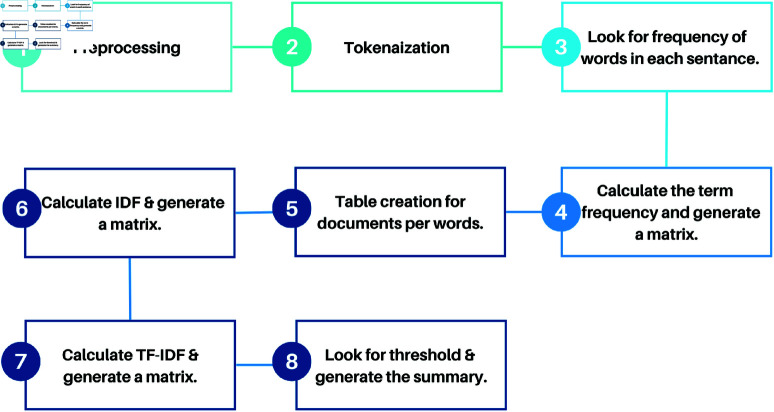
TF-IDF (term frequency-inverse document frequency).

At first, we used Term Frequency-Inverse Document Frequency (TF-IDF) vectorization shown in Fig 7 to turn preprocessed Bangla text into numerical vectors. This method helped us show how important words are within a document. After that, TF-IDF assigns weights to words based on their frequency in a document and their rarity across all documents. This creates a sparse matrix that highlights unique terms for each news category, eventually reducing the impact of common words that do not result in much information gain. In our work, we used scikit-learn’s TfidfVectorizer with a maximum of 30,000 features. We looked at both single words and pairs of words (unigrams and bigrams). This approach works particularly well for the support vector machine (SVM) which is part of our stacking classifier.

#### 3.3.2 Sequential features.

To capture the sequential nature of text and long-range dependencies, we used a Bidirectional Long Short-Term Memory (BiLSTM) network.

- **Tokenization:** Before feeding the text into the model, We used TensorFlow’s Tokenizer to convert the cleaned text into sequences of integers. Upon further analysis we found 443,759 rare words that occur only once. These extremely rare words were essentially “noise" in the data - they didn’t add much value to understanding the overall content or helping with classification. Thus choosing 30,000 words as a base number for our max feature which accounted for 99.24% of the total word occurrences. The vocabulary was limited to the top 30,000 most frequent words (max_features = 30000), with an oov_token to handle out-of-vocabulary words.
- **Sequence Padding:** The tokenized text was transformed into sequences of integers and padded to a fixed length of 399 tokens using TensorFlow’s pad_sequences. Padding ensured uniform input dimensions for the BiLSTM model.
- **Embedding Layer:** The padded sequences were passed through an embedding layer with a dimensionality of 300, which generated dense vector representations for each token, capturing semantic relationships between words. The study employed this embedding layer to capture rich semantic and syntactic relationships among Bangla words. These embeddings were learned directly during training as part of the BiLSTM model. After careful experimentation with other embedding techniques, including pre-trained embeddings, this approach was found to be more effective for the hybrid model.

Then, two Bidirectional LSTM layers process these embedded sequences, extracting all the temporal features and also maintaining the long-term dependencies which is crucial for understanding the context in news articles. In addition, a global max pooling layer follows, reducing dimensionality by selecting key features across the sequence. Finally, multiple dense layers with ReLU activation further process the features, creating high-level representations of news content, with dropout applied between layers to prevent overfitting. This sequential feature extraction method is particularly effective in capturing contextual information and specific language patterns in Bangla news articles.

#### 3.3.3 Meta-features.

Our stacking approach generates meta-features by combining outputs from multiple base classifiers. The TF-IDF-based SVM model produces probability distributions across news categories for each article, while the BiLSTM model generates predictions based on its deep sequential understanding of the text. These intermediate predictions are merged to create a set of meta-features, generating a combination of probabilistic insights with deep sequential understanding. Finally, these meta-features serve as input for the final meta-model, which is a neural network that learns to interpret and combine outputs from both base models. The meta-model consists of several dense layers with dropout for regularization, which allows it to learn complex relationships between the predictions of the base models.

#### 3.3.4 Feature integration.

The diverse feature sets described above are integrated through our stacking classifier approach. This multi-level feature extraction process helped us to transform raw Bangla news text into sophisticated mathematical representations, eventually, capturing both fine-grained lexical information and high-level semantic patterns. By combining statistical methods (TF-IDF with SVM), deep learning techniques (BiLSTM), and a neural network meta-model, our BanglaNewsClassifier achieves a comprehensive understanding of news content. This rich feature set enables nuanced distinctions between different news categories in the Bangla language, contributing significantly to the high performance of our classification system.

#### 3.3.5 Deep learning embeddings.

For our BiLSTM model, we employ word embeddings to convert words into dense vector representations. The embedding layer learns to represent each word as a fixed-length vector of 300 dimensions. These vector representations encode semantic and syntactic information about the words, allowing our models to understand relationships between words in the Bangla language. In the BiLSTM model, these learned embeddings serve as the initial representation for each word in the sequence. This allows the BiLSTM to process the text while learning and maintaining rich semantic information specific to our classification task.

### 3.4 Classifiers

During our research on Bangla news classification, we experimented with several machine learning models and got a wide range of performances. The whole experimentation process can be divided into three categories. Traditional Machine learning models, Deep Learning models, and Hybrid models.

#### 3.4.1 Traditional machine learning models

In our text classification research, we started with logistic regression (LR), which did not meet our accuracy goals. Naive bayes performed poorly due to high bias, and random forest, while strong on training data, also struggled with overfitting on the test set.

1. **Logistic Regression (LR):** Logistic regression is a fundamental statistical method employed for binary classification tasks. It models the probability of an outcome as a function of input features, utilizing the logistic function to constrain predictions between 0 and 1 [26]. This technique finds widespread application in natural language processing for tasks such as sentiment analysis and text categorization.
  $$\hat{y}=\frac{1}{1+e^{-(𝐰\cdot 𝐱+b)}}$$ (1)
  Eq (1) refers to the logistic functions. Here, $\hat{y}$ is the predicted probability, *w* is the weight vector,*x* is the feature vector, and *b* is the bias term.
  $$L=-\frac{1}{N}\sum_{i=1}^{N}\left[y_{i}\log({\hat{y}}_{i})+(1-y_{i})\log(1-{\hat{y}}_{i})\right]$$ (2)
  Here, Eq (2) refers to cross-entropy loss. Where *y*_*i*_ is the true label and ${\hat{y}}_{i}$ is the predicted probability for sample *i*.
2. **Naive Bayes (NB):** Naive bayes is a probabilistic classification algorithm based on Bayes’ theorem with an assumption of independence among predictors. It works with posterior probability calculations for given classes and selects the outcome with the highest probability [27]. Even after being simple and “naive" assumptions, this method has proven effective in various text classification tasks, including spam detection and document categorization.
  $$P(C_{k}|𝐱)=\frac{P(C_{k})\prod_{i=1}^{n}P(x_{i}|C_{k})}{P(𝐱)}$$ (3)
  Here, $P(C_{k}|𝐱)$ in Eq 3 refers to the posterior probability of class *C*_*k*_ given features *x*,*P*(*C*_*k*_) is the prior probability of class *C*_*k*_, $P(x_{i}|C_{k})$ is the prior probability of class *C*_*k*_, and P(𝐱) is the evidence of marginal likelihood of the feature vector *x*.
3. **Random Forest (RF):** Random forest is an ensemble learning method that constructs multiple decision trees during the training phase and outputs the class that is the mode of the classes (classification) or mean prediction (regression) of the individual trees [28]. For its high accuracy, ability to handle large datasets, and resistance to overfitting, this algorithm is well known.
  $$\hat{y}=\text{mode}(\{T_{1}(𝐱),T_{2}(𝐱),\ldots ,T_{M}(𝐱)\})$$ (4)
  $T_{m}(𝐱$ in the Eq (4) refers to the prediction of the *m*–*th* tree for the input *x*, *M* is total numbers of trees and *haty* is the predicted class(majority vote among all trees).
4. **Decision Tree (DT):** Decision tree recursively splits the dataset based on feature values to classify data through supervised learning [29]. At each node, a split is made to minimize impurity, using measures like Gini Impurity in Eq 5 or Entropy in Eq 6:
  $$Gini(D)=1-\sum_{i=1}^{n}p_{i}^{2}$$ (5)
  $$Entropy(D)=-\sum_{i=1}^{n}p_{i}\log_{2}(p_{i})$$ (6)
5. **Support Vector Machine(SVM):** SVM is a powerful classifier, it maximizes the margin between classes to find the optimal hyperplane to separate data points [30]. SVM is effective in high-dimensional spaces but can be computationally intensive. For linearly separable data, the optimization is:
  $$\min_{𝐰,b}\frac{1}{2}\|𝐰{\|}^{2}$$ (7)
  $$y_{i}(𝐰\cdot {𝐱}_{i}+b)\geq 1$$ (8)
  $$K({𝐱}_{i},{𝐱}_{j})=\exp(-\gamma \|{𝐱}_{i}-{𝐱}_{j}{\|}^{2})$$ (9)

Here, Eq 7 is an objective function, subject to Eq 8. In addition, w is the weight vector, b is the bias term, *x*_*i*_ are the feature vectors, and $y_{i}\in \{-1,1\}$ are the class labels. In addition, for non-linear data, SVM uses kernel functions like the RBF kernel shown in Eq 9. Here γ is a free parameter.

#### 3.4.2 Deep learning models.

As the traditional machine learning models did not meet our expectations, we turned to deep learning techniques like CNN, and LSTM. At first, LSTM showed promise with its ability to handle sequential text. Then we ran our dataset with CNN and it outperformed all previous models, achieving our highest accuracy by effectively capturing local patterns in the data

1. **CNN:** Convolutional Neural Networks (CNNs) are deep learning architectures primarily used for processing grid-like data, such as images. They employ convolutional layers to automatically and adaptively learn spatial hierarchies of features [31]. Computer vision tasks have also been successfully applied to natural language processing with the help of CNNs.
  $$y_{i,j,k}=\sum_{m=1}^{M}\sum_{n=1}^{N}\sum_{c=1}^{C}x_{i+m-1,j+n-1,c}\cdot w_{m,n,c,k}+b_{k}$$ (10)
  In Eq (10), *y*_*i*,*j*,*k*_ represents the output at position (i,j) for the *k*–*th* filter in the convolutional layer. Input value from the c-th channel of the input feature map at position(i+m,j+n-1) in repressed by this *x*_*i* + *m*−1,*j* + *n*−1,*c*_ and *w*_*m*,*n*,*c*,*k*_ refers to the weight of the filter.
2. **LSTM:** Long Short-Term Memory (LSTM) networks are a type of recurrent neural network designed to learn long-term dependencies [32]. They incorporate memory cells and gating mechanisms to selectively remember or forget information over long sequences. When it comes to sequential data tasks, including language modeling and machine translation LSTM’s are widely used.
  $$h_{t}=W_{o}h_{x}+b_{o}\cdot \tanh(W_{f}h_{x}+b_{f}C_{t-1})+W_{i}h_{x}+b_{i}\cdot \tanh(W_{C}h_{x}+b_{C})$$ (11)
  Here, to make Eq 11 more concise and easy to understand we have taken $h_{x}=[h_{t-1},x_{t}]$. To explain further, *h*_*t*_ in the equation refers to the current hidden state(output at time step t) and *x*_*t*_ refers to the input at the current time step t. Others are as follow, *h*_*t*−1_: previous hidden state(from time step t-1), *C*_*t*−1_: previous cell state, tanh(): activation function. Finally, *W*_*o*_, *W*_*i*_, *W*_*f*_, *W*_*C*_ refer to weight matrices for output, input, forget gates, and cell update respectively and *b*_*o*_, *b*_*i*_, *b*_*f*_,*b*_*C*_ are bias terms for each gate.
3. **Hybrid models: Stacking classifiers:** Initially, we evaluated traditional machine learning models such as logistic regression, naive bayes, random forest, and support vector machines (SVM). Their performance served as a baseline to compare against advanced models. Then we roam around the deep learning arc Deep learning architectures, including CNN and BiLSTM. After surveying the top performers we combined different architectures to leverage their complementary strengths. First, we combined naive bayes with CNN, but the results were disappointing. Next, we paired CNN and LSTM, using CNN for feature extraction and LSTM for their ability to effectively capture spatial hierarchies in text data, enhancing feature extraction for improved accuracy [33].
  **CNN+LSTM:** In this setup, the CNN first processes the input data, and its output serves as the input for the LSTM. Formally, we can express this process through the following equations
  $$y_{\text{CNN}}=\text{CNN}(x)$$ (12)
  $$h_{t}=\text{LSTM}(y_{\text{CNN}})$$ (13)
  Combining Eq 12 and Eq 13, we get:
  $$\hat{y}=f(W_{\text{out}}\cdot h_{t}+b_{\text{out}})$$ (14)
  Here, $W_{\text{out}}$ in the Eq 14 is the weight matrix for the output layer and $b_{\text{out}}$ is the bias term for the output layer. Finally, the equation as a whole looks like Eq 15.
  $$\hat{y}=f(W_{\text{out}}\cdot \text{LSTM}(\text{CNN}(x))+b_{\text{out}})$$ (15)
  Stacking classifiers were chosen for this study because they excel in leveraging the strengths of heterogeneous models, making them particularly effective for the imbalanced Bangla News dataset. Unlike other ensemble techniques, stacking employs a meta-learner to combine predictions from diverse base models, enabling it to address the dataset’s specific challenges, such as category overlap and varying class distributions. This limitation reduces its ability to generalize across categories with highly variable feature distributions. Boosting adjusts mistakes by giving more importance to incorrectly classified instances. This helps with balanced datasets. But for imbalanced datasets like ours, problems arise. “Technology" (8.4%) has fewer examples compared to “economic" (15.8%) or “entertainment" (14.3%). Boosting sometimes focuses too much on errors in small classes, causing instability. Overlapping categories like “education" and “technology" may get classified wrongly. Also, boosting’s step-by-step process can increase noise in data, hurting performance for unclear categories. Soft voting classifiers combining BiLSTM and CNN predictions were also tested. The results were not as good. Voting classifiers use average probabilities or majority votes to make decisions. These lack the smart decision-making layer in stacking. Thus, they found it difficult to sort out conflicting predictions for similar categories, such as “state" and “politics" or “economic" and “technology". Stacking was selected because it smartly combines the strengths of different models. It beats the limits of bagging, boosting, and voting classifiers. Our stacking classifier mixes BiLSTM, useful for “economics" and “sports," with SVM, great at drawing decision lines in complex spaces, especially in overlapping classes like “state" and “politics." The meta-learner looks at the probabilistic outputs of base models. This approach weakens their solo downsides and focuses on their complementary strengths. To illustrate, the local feature extraction capabilities of CNN with the combination of BiLSTM’s long-term sequence modeling power the stacking classifier worked really well. Moreover, stacking classifiers helps with generalization by averaging out the errors of individual models, which in our case helped us to reduce the risk of poor performance due to the limitation of a single model. Finally, it can be said that these two models complemented each other’s strengths mitigated each other’s weaknesses, and worked as a more robust and effective classification model.
  **Meta-stacking classifier:** Finally, we used an ensemble model for Bangla text classification that combines support vector machines (SVM) and Bidirectional Long Short-Term Memory (BiLSTM) networks. The model can be expressed as:
  $$y=f_{\text{meta}}(f_{\text{SVM}}(x),f_{\text{BiLSTM}}(x))$$ (16)
  Here, $f_{\text{meta}}$ in the Eq 16 is the meta-classifier, and $f_{\text{SVM}}$ and $f_{\text{BiLSTM}}$ are the base classifiers. This stacking approach aims to reduce bias and variance by leveraging the strengths of both linear and sequential models. We split the data by an 80 by 20 ratio. Then we encoded the labels, sequentially the text data was tokenized. We also set the maximum padded length to merely 399 tokens. Furthermore, the vocabulary size was limited to merely 30,000. For the SVM component, implemented using sklearn’s Pipeline, the TF-IDF vectorization can be represented as:
  $$X_{\text{tf-idf}}=\text{TF-IDF}(\text{unigrams},\text{bigrams})$$ (17)
  This Eq 17 transforms the text input into feature vectors. The SVM then applies an RBF kernel with Eq 18:
  $$K(x_{i},x_{j})=\exp\left(-\gamma \|x_{i}-x_{j}{\|}^{2}\right)$$ (18)
  Here,$\gamma =\frac{1}{2\sigma^{2}}$, with C = 1.0 and γ=`scale'.
  The BiLSTM model was built with the TensorFlow library and includes an embedding layer of 300 dimensions shown in 19:
  $$X_{\text{embed}}=\text{Embedding}(𝐗,\text{300 dimensions})$$ (19)
  The BiLSTM layers are defined in Eq 20 as:
  $$h_{\text{BiLSTM}}=\text{BiLSTM}({𝐗}_{\text{embed}})$$ (20)
  With the first BiLSTM layer having 128 units and the second having 64 units. A Global Max Pooling layer, followed by two dense layers with 128 and 64 units using ReLU activation and dropout rates of 0.5 and 0.3, respectively, follows. The meta-classifier is a neural network that operates on the concatenated outputs of the SVM and BiLSTM models. The architecture of the meta-classifier is shown in Eq 21:
  $$h_{\text{meta}}=\text{De}(256,\text{Re})\to \text{Dr}(0.5)\to \text{De}(128,\text{Re})\to \text{Dr}(0.3)\to \text{De}(64,\text{Re})\to \text{Dr}(0.2)$$ (21)
  Here, De = Dense, Re = ReLU, Dr = Dropout.
  The final output layer uses softmax activation in Eq 22 for multiclass probability output:
  $$\hat{y}=\text{softmax}({𝐡}_{\text{meta}})$$ (22)
  In the training process, the SVM and BiLSTM models are first trained independently on the training data. Both models then generate probability predictions $p_{\text{SVM}}$ and $p_{\text{BiLSTM}}$ for the training and test sets in Eq 23, which are concatenated to form meta-features:
  $$p_{\text{meta}}=[{𝐩}_{\text{SVM}},{𝐩}_{\text{BiLSTM}}]$$ (23)
  The meta-classifier is subsequently trained on these meta-features. Optimization uses the Adam optimizer with an initial learning rate of α=0.001 for both the BiLSTM and the meta-classifier. To address overfitting, dropout layers (rates: 0.5 and 0.3) were used in the BiLSTM and meta-classifier, and early stopping with patience of 5 epochs was applied to prevent excessive training. Also, Underfitting was mitigated through hyperparameter tuning, such as optimizing embedding dimensions (300), BiLSTM units (128, 64), and kernel parameters in SVM (RBF kernel with $\gamma {=}^{\prime }scale^{\prime }$). And, the dataset size (118,404 articles) is sufficiently large to reduce the risk of underfitting.
  Our extensive tests on hyperparameters showed specific configurations that regularly gave the best mix of accuracy and generalization. These optimal settings reduced overfitting and improved performance in every category. They tackled the problems of the dataset’s uneven distribution and overlapping categories. Table 2 shows the chosen hyperparameters.

**Table 2:** Hyperparameters for different models and components.

Model/Component	Hyperparameter	Value
	max_features	30000
	ngram_range	(1, 2)
SVM Classifier	kernel	rbf
	C	1.0
	gamma	scale
Tokenizer	num_words	30000
Padding	max_len	399
	batch_size	128
LSTM Model	dropout_rate_1	0.5
	dropout_rate_2	0.3
	loss	sparse_categorical_crossentropy
	dropout_rate_1	0.5
Meta Model	dropout_rate_2	0.3
	dropout_rate_3	0.2
	early_stopping_patience	5
Callbacks	reduce_lr_factor	0.2
	reduce_lr_patience	3

### 3.5 Experimental setup

In our experimental setup, we simulated environment and runtime configuration to ensure reproducibility and seamless performance across different environments using Docker Studio. We conducted the fine-tuning process on our local machine consisting of an NVIDIA RTX 3060 Ti (8GB VRAM), an AMD Ryzen 7600X processor, and 32GB of RAM. To avoid library conflicts, we set up a Colab-compatible runtime environment using Docker. We established a controlled software environment that mirrors cloud-based configurations while taking full advantage of our local hardware. This containerization not only isolates dependencies and ensures consistency across runs but also facilitates easier scalability and benchmarking across different platforms. Within this environment shown in Table 3, our software stack included Ubuntu 22.04, TensorFlow 2.x, Keras, and scikit-learn. Firstly, in the fine-tuning process, we fixed seeds for NumPy then we configured GPU memory growth to dynamically allocate memory on the GPU as needed, thereby avoiding potential memory allocation issues on our GPU.

**Table 3:** System specifications and configuration.

Category	Component	Specification / Configuration
Hardware	GPU	NVIDIA RTX 3060 Ti (8GB VRAM)
	CPU	AMD Ryzen 7600X
	Memory (RAM)	32GB
Software Environment	Operating System	Ubuntu 22.04
	Containerization	Docker (via Docker Studio with a Colab-compatible runtime)
Software Stack	TensorFlow	2.18.0
	scikit-learn	Version 1.2
Fine-Tuning Process	Reproducibility	Fixed seeds for NumPy
	GPU Memory Management	Configured GPU memory growth (dynamic allocation)

## 4 Results and discussion

Our machine learning models for the classification of Bangla news articles showed significant variations in performance, each highlighting different strengths and weaknesses.

We evaluated our models using accuracy in Eq 24, precision in Eq 25, recall in Eq 26, and F1 score in Eq 27 and provided a comprehensive view of their performance. The equations for these metrics are:

$$Accuracy=\frac{\left(TP+TN\right)}{\left(TP+TN+FP+FN\right)}$$ (24)

$$Precision=\frac{TP}{\left(TP+FP\right)}$$ (25)

$$Recall=\frac{TP}{\left(TP+FN\right)}$$ (26)

$$F1=\frac{2*Precision*Recall}{Precision+Recall}$$ (27)

Where TP=TruePositives, TN=TrueNegatives, FP=FalsePositives, and FN=FalseNegatives.

**Fig 8:**
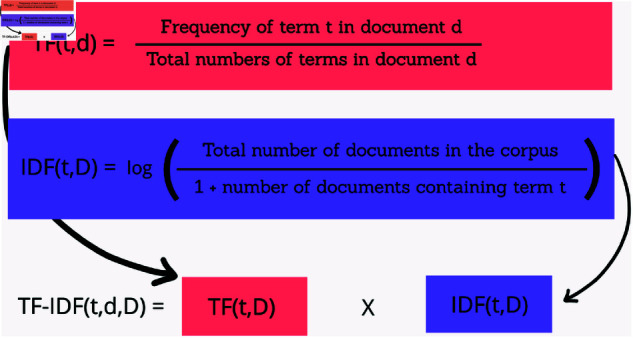
Confusion matrix of (a) CNN and (b) LSTM.

The confusion matrices reveal intriguing patterns in Fig 8(a) how CNN and LSTM in Fig 8(b) models handle news classification. While both excel at identifying political and entertainment content, they struggle with state-related news, often confusing it with political articles. This suggests a significant overlap in language and themes between state and political news, posing a challenge for automated classification systems. The CNN model demonstrates a particular strength in recognizing sports-related content, possibly due to its ability to capture local features in text that are indicative of sports reporting. Conversely, the LSTM model shows a nuanced understanding of economic news, outperforming CNN in this category. This could be attributed to the LSTM’s capacity to maintain long-term dependencies, which may be crucial for grasping complex economic narratives. Interestingly, both models exhibit difficulty in cleanly separating technology news from other categories, especially state news, hinting at the pervasive nature of technology-related topics across various news domains.

**Fig 9:**
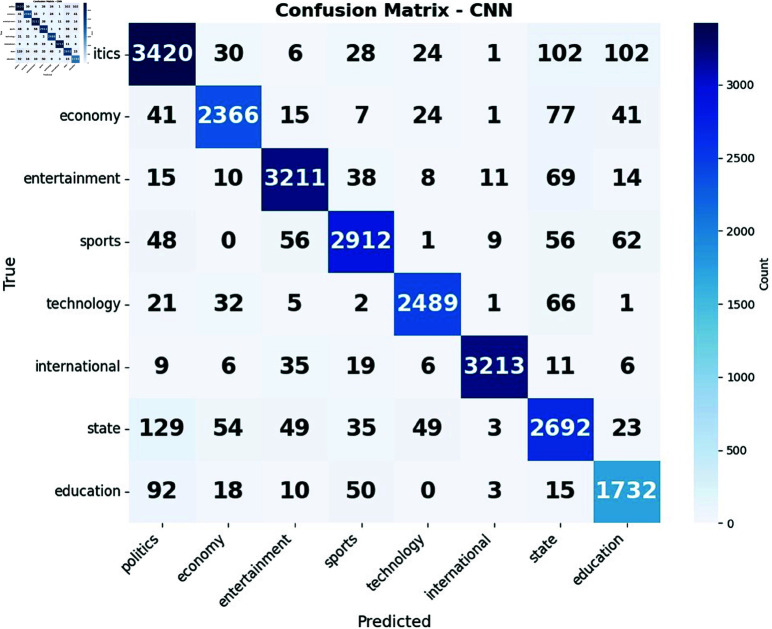
Confusion matrix of (a) Decision Tree and (b) Logistic Regression.

The confusion matrices presented in Figs 9(a) and 9(b) elucidate the performance of decision tree and logistic regression models in classifying news articles across eight distinct categories. Both models exhibit strong diagonal elements, indicative of generally accurate predictions. However, the logistic regression model demonstrates superior precision in classifying politics and economy-related articles, while the decision tree model excels in categorizing sports and international news. These disparities highlight the varying strengths of each algorithm in capturing the nuanced features of different news domains.

A notable pattern emerges in both models regarding the persistent misclassification between the “state" and “politics" categories. This consistent confusion suggests a significant overlap in the linguistic features or thematic content of state and political news articles. Such a finding underscores the inherent challenge of distinguishing between these closely related categories in automated news classification systems. This observation points to the potential need for more sophisticated feature engineering techniques or the implementation of hierarchical classification approaches to better delineate the subtle distinctions between state and political news content. The recurrence of this misclassification across both models emphasizes that this is not merely an algorithm-specific issue but rather a fundamental challenge in the domain of news article categorization.

**Fig 10:**
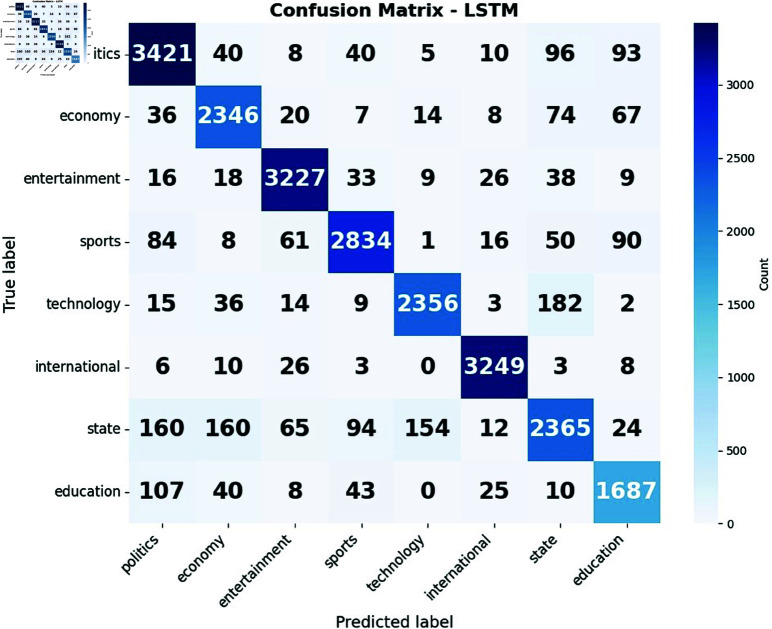
Confusion matrix of (a) Naive Bayes and (b) Random Forest.

The confusion matrices for naive bayes in Fig 10(a) and for random forest in Fig 10(b) reveal unique patterns in their performance for news classification. We found that Random Forest excels in classifying economy related news compared to naive bayes. Conversely, naive bayes demonstrates a particular strength in identifying international news, outperforming random forest in this category. Both models struggle with the technology category, often misclassifying it as economy-related content, indicating a potential overlap in terminology between these domains. The state category poses challenges for both models, with frequent misclassifications as politics, highlighting the difficulty in distinguishing between state and national political news. Interestingly, random forest shows a more balanced performance across categories, while naive bayes shows more noticeable changes in accuracy between different news types. These small differences in performance across categories highlight the importance of choosing the right model based on the specific needs of the news classification task.

**Fig 11:**
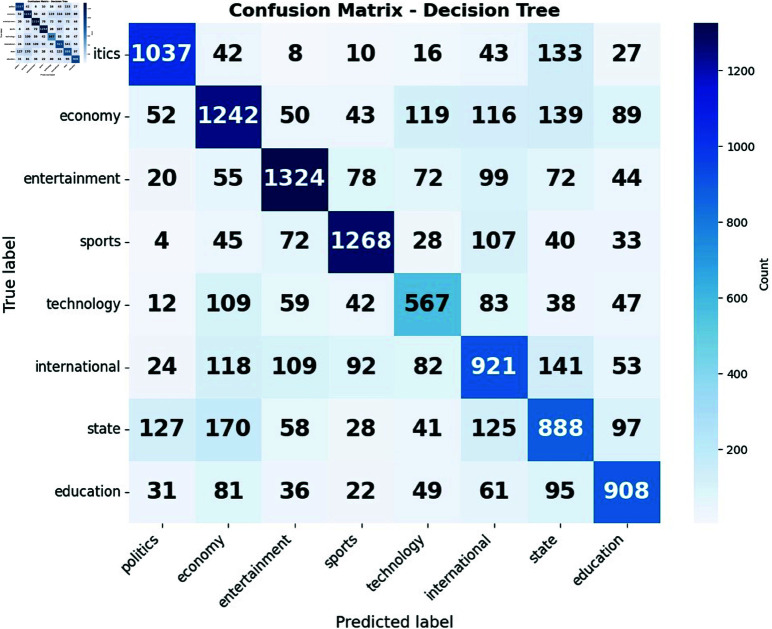
Confusion matrix of (a) Ensemble and (b) Stacking Classifier.

Based on their performance, we categorized our models into three groups: “Top Performers," “Moderate Performers," and “Low Performers."

The stacking classifier that combines LSTM and SVM emerged as the top performer, boasting an impressive 94% accuracy on the test set shown in Table 4. This remarkable achievement can be attributed to the synergistic relationship between LSTM and SVM, each bringing its unique strengths to the table. LSTMs excel at handling sequential data, effortlessly capturing temporal dependencies and long-range patterns within text. Meanwhile, SVMs thrive in high-dimensional spaces and possess an uncanny ability to pinpoint the optimal hyperplane that maximizes the margin between classes. In this clever stacking arrangement, LSTM serves as a powerful feature extraction tool, meticulously preparing the data for SVM to work its magic in determining the text class. This harmonious collaboration not only compensates for each model’s weaknesses but also significantly reduces the risk of overfitting while enhancing overall generalization. The result is a robust and versatile classifier that leverages the best of both worlds, demonstrating the immense potential of hybrid approaches in tackling complex natural language processing tasks.

**Table 4:** Performance analysis of different combinations of models in our dataset.

Model	Accuracy	Precision	Recall	F1 Score
NB [27]	0.84	0.85	0.84	0.83
DT [29]	0.69	0.69	0.69	0.68
RF [28]	0.86	0.87	0.86	0.86
LR [34]	0.86	0.86	0.86	0.86
CNN [35]	0.93	0.93	0.93	0.93
LSTM [32]	0.93	0.93	0.93	0.93
CNN + LSTM [33]	0.93	0.93	0.93	0.93
**Proposed (BiLSTM + SVM)**	**0.94**	**0.94**	**0.94**	**0.94**

In the training process, we found that logistic regression performed moderately well, achieving test accuracies around 89-90%. These models, when combined with TF IDF, proved to be effective for Bangla news classification. The naive Bayes model, despite its assumption of feature independence, performed reasonably well with an accuracy of 85%. This suggests that while the independence assumption may not hold strictly for news text, it still provides a useful approximation for classification purposes. The random forest model had a testing accuracy of (86%) which is almost similar to logistic regression. Random forests are better at handling the high dimensionality and potential noise in text data,

The Soft Voting Ada Boost + CNN Voting Classifier underperformed compared to other models. This can be explained by the limitations of soft voting when base models are not well-suited to the classification task. If the base models make similar errors, averaging their predictions in soft voting won’t correct those errors. If we represent the predictions of each model as p1, p2, ..., pn, soft voting calculates the final prediction as:

$$\text{Final Prediction}=\arg\max_{1\leq i\leq n}p_{i}(c|x)$$ (28)

Models such as KNN and Decision Tree underperformed significantly in this text classification task. KNN struggles with high-dimensional data, which is typical in text classification problems. As the number of dimensions increases, the volume of the space increases exponentially. This means that data points become sparse and distant from each other in high-dimensional spaces. In text classification, where each unique word can be considered a dimension, the number of dimensions can easily reach thousands or even tens of thousands. Similarly, the decision tree model faces its own challenge: The decision tree model achieved only 68% accuracy while random forest Archived 86% accuracy. This substantial difference can be attributed to decision tree models that tend to overfit because they memorize specific patterns, noise, or outliers present in a dataset.

ROC curves are used alongside confusion matrices for performance evaluation and provide a dynamic view of classifier performance. While confusion matrices offer a snapshot of performance at a single decision threshold, ROC curves illustrate the trade-off between true positive rate and false positive rate across all possible thresholds. This representation is particularly valuable in multi-class classification scenarios, such as news categorization, where optimal threshold selection may vary across categories and use cases. In this particular case, ROC curves and their associated Area Under the Curve (AUC) metrics offer a threshold-independent measure of classifier performance which is especially beneficial when dealing with the costs of false positives and false negatives differ across categories, situations common in news classification tasks.

**Fig 12:**
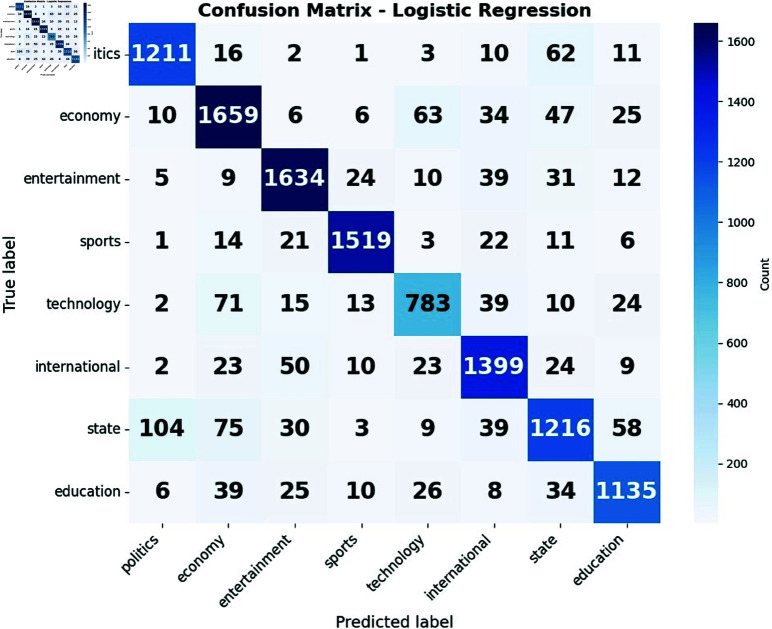
ROC curve of (a) CNN and (b) LSTM.

**Fig 13:**
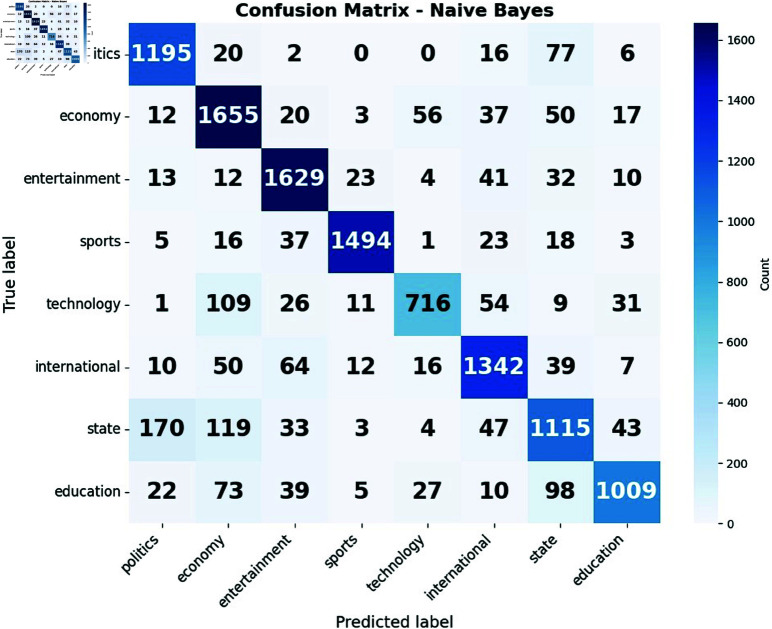
ROC curve of (a) Decision Tress and (b) Logistic Regression.

**Fig 14:**
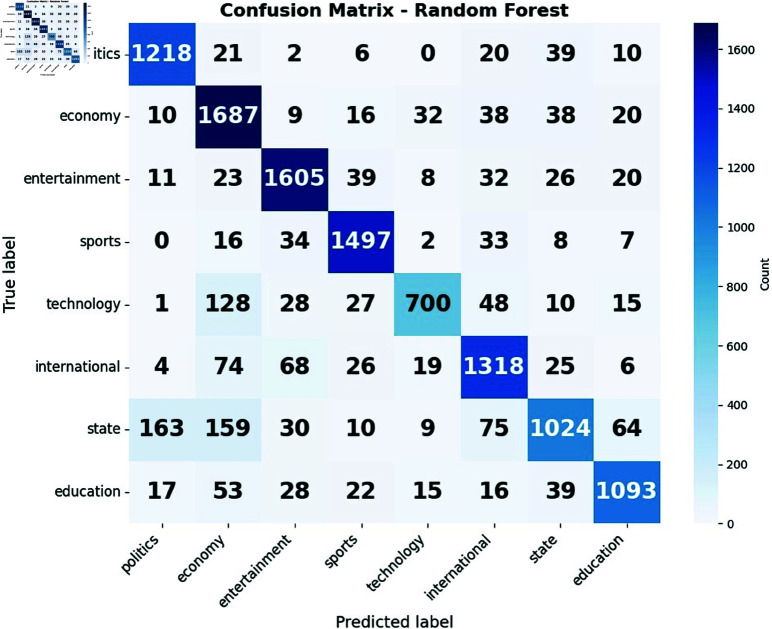
ROC curve of (a) Naive Bayes and (b) Random Forest.

**Fig 15:**
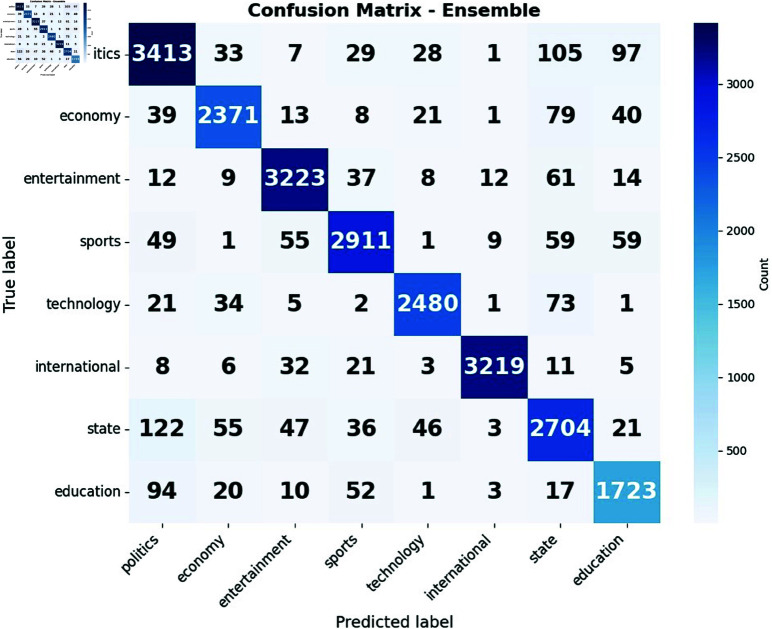
ROC curve of (a) Ensemble and (b) Stacking Classifier.

There is a total of eight ROC curves. Two were created for deep-learning models, four were for machine-learning models and two were for hybrid models. The models are CNN in Fig 12(a), LSTM in Fig 12(b), decision tree in Fig 13(a), logistic regression in Fig 13(b), naive bayes in Fig 14(a), and Random Forest in Fig 14(b). Also, the ROC for two hybrid models Ensemble (CNN+LSTM) and Stacking Classifier (BiLSTM + SVM) is shown in Fig 15(a) and Fig 15(b), respectively. The curves show performance across various news categories. Categories include politics, economy, entertainment, sports, technology, international affairs, state news, and education. The visualizations reveal performance differences between news types and models. The ‘state’ category consistently shows lower performance (lower AUC values) across models. This suggests that state news is more challenging to classify accurately. ‘Sports’ and ‘international’ categories often perform better (higher AUC values). This indicates these categories have more distinct features for classification. LSTM, CNN, and logistic regression models show strong overall performance. These models have smooth, high-arching ROC curves. Their curves approach the top-left corner of the plot. This suggests robust classification capabilities for these models. Such performance is crucial for effective news categorization in real-world applications.

To gain deeper insights into our models’ performance, we conducted an error analysis focusing on misclassifications: Confusion Matrix: Analysis of the confusion matrix for our best-performing stacking classifier revealed that political news was often misclassified as economic news, likely due to overlapping vocabulary and themes of the topic. Similarly, technology news was sometimes confused with educational news, possibly due to shared technical terminology. Also sometimes they share similar themes as these topics are highly correlated. Misclassification examples:

1. A news article about a government policy on technology education was misclassified as “technology” instead of “education.” This highlights the challenge of distinguishing between closely related categories.
2. An entertainment news piece discussing the economic impact of a film festival was incorrectly classified as “economic" news, demonstrating the model’s sensitivity to economic terms even in non-economic contexts.

After reviewing some of the samples from the misclassified sections we found that some of the articles indeed share two or three themes and they could hardly be classified in one class. Given that many articles span multiple themes, transitioning to a multi-label classification model would be more appropriate.

**Fig 16:**
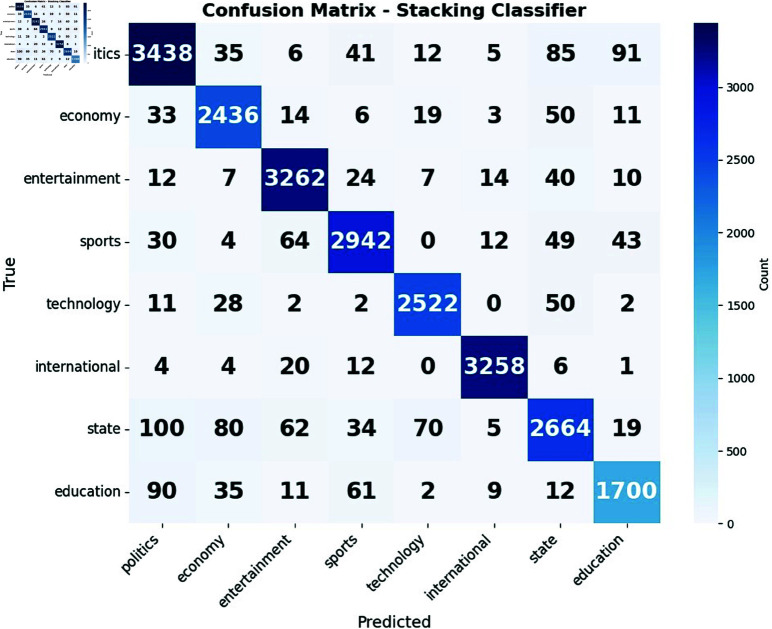
Some highlighted words form word-cloud.

For our traditional machine learning models, we analyzed feature importance to understand which words or n-grams were most influential in classification decisions: Using mutual information scores, we identified the top features for each category. For example:

We made word clouds for each news category. These clouds show in Fig 16 which words were most important for telling categories apart. The biggest words in each cloud were the ones that helped the computer recognize that category best. This helped us see what kinds of words are typically used in different types of news stories.

We observed a general trend where more complex models like our stacking classifiers and deep learning models outperformed simpler models. However, this came at the cost of increased complexity and more resources being used. For instance:

- The stacking classifier with a combination of SVM and LSTM achieved the highest accuracy (94%) but required the most computational resources and training time.
- The CNN model offered a good balance between performance (93.04% accuracy) and computational efficiency.
- Simpler models like naive bayes were much faster to train but sacrificed some accuracy (85%).

This trade-off between model complexity and performance highlights the importance of considering both accuracy and computational efficiency when choosing a model for real-world applications. In conclusion, our experiment shows that ensemble methods (especially stacking classifiers) and deep learning models (LSTM and CNN) perform very well in Bangla news article classification. Traditional machine learning models also perform moderately well. But models like KNN and decision tree struggled with the complexity and high dimensionality of Bangla text data and hence performed poorly. Also, it shows that we should choose the right models and the right ensemble techniques if we want to get satisfactory results.

## 5 Conclusion and future work

Throughout the study we tried to address critical gaps in natural language processing for low-resource languages, eventually achieving significant advancement in the field of Bangla news classification. Through our comprehensive approach which includes data collection, preprocessing, and implementation of various machine learning and deep learning models, we achieved valuable insights and promising results. Our stacking meta-classifier (BiLSTM + SVM) performed the best with an F1 score of 0.94 which is better than all other traditional machine learning models in complex text classification. Also, key findings are deep learning models (CNN and BiLSTM) can capture the pattern of Bangla text, and traditional machine learning algorithms (naive bayes, decision trees, random forests, and logistic regression) are important. The study highlights the importance of sophisticated feature extraction methods, including TF-IDF vectorization and learned embeddings, in representing the nuances of the Bangla language. However, in some cases we lacked, such as the dataset collected from specific Bangla newspapers, it is unable to represent the diversity of Bangla news language and style. Also, the Complexity of hybrid models. For instance, BiLSTM + SVM needs a good amount of computational power and training time. Moreover, through our study we open the gates for future researchers to explore advanced architectures, addressing imbalance datasets and refining classification for closely related categories. Also, they can increase the diversity of the news articles with open-source Bangla new articles or buy copy-written articles with funding and also try to reduce computational overhead. This finding contributes to Bangla natural language processing along with valuable methodologies applicable to text classification in other low-resource languages. It also opens the gates for enhancing information retrieval and context management systems in Bangla-speaking regions. As we continue to refine these techniques, we anticipate further improvements in accuracy and efficiency, ultimately contributing to the broader goal of making advanced NLP technologies more accessible and effective for low-resource languages.
