# Correction: BanglaNewsClassifier: A machine learning approach for news classification in Bangla Newspapers using hybrid stacking classifiers

**DOI:** 10.1371/journal.pone.0332710

**Published:** 2025-09-17

**Authors:** Tanzir Hossain, Ar-Rafi Islam, Md Humaion Kabir Mehedi, Annajiat Alim Rasel, M. Abdullah-AL-Wadud, Jia Uddin

Figs 3–16 were uploaded incorrectly. Please see the correct Figs 3–16 here.

**Fig 3:**
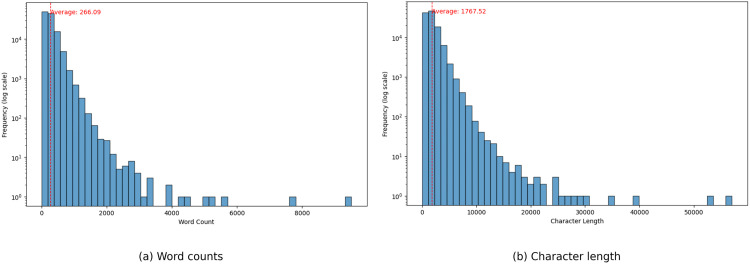
Distribution of word counts and character length.

**Fig 4:**
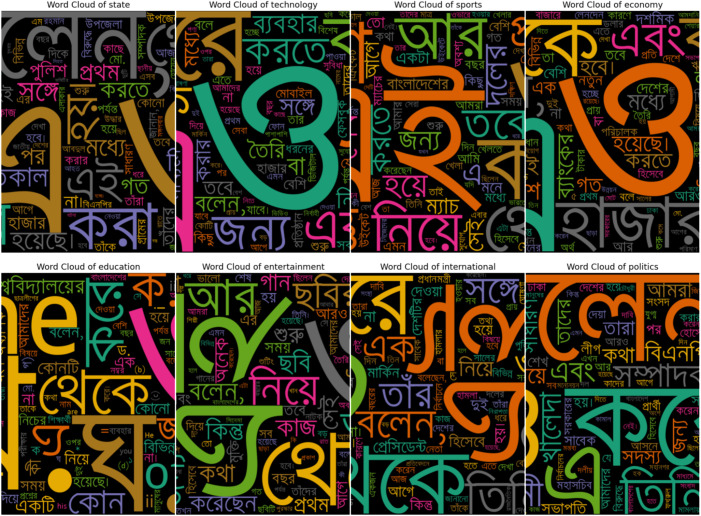
Word cloud before pre-processing.

**Fig 5:**
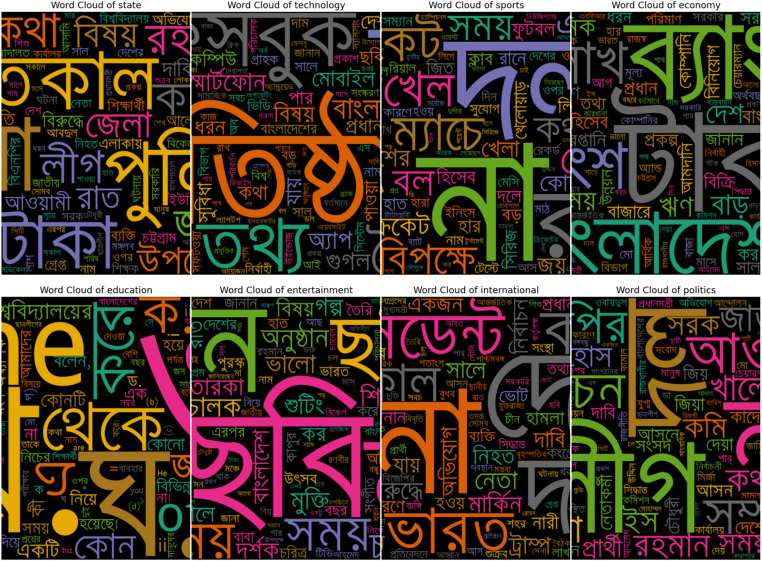
Word cloud after pre-processing.

**Fig 6:**
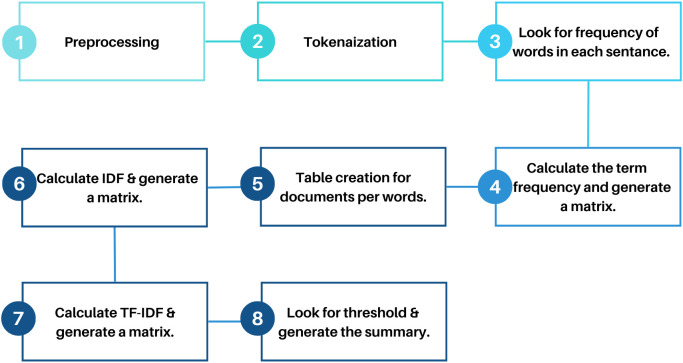
Global feature extraction process.

**Fig 7:**
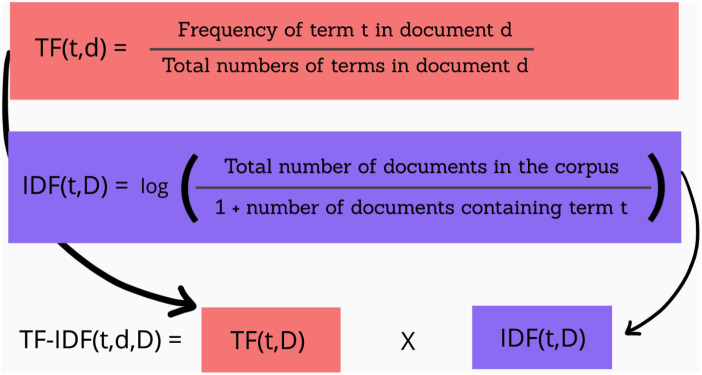
TF-IDF (Term Frequency-Inverse Document Frequency).

**Fig 8:**
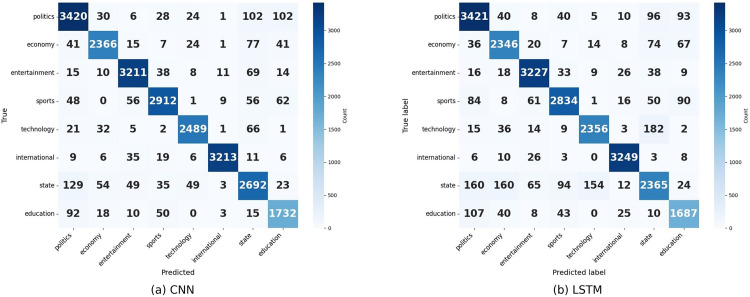
Confusion matrix of CNN and LSTM.

**Fig 9:**
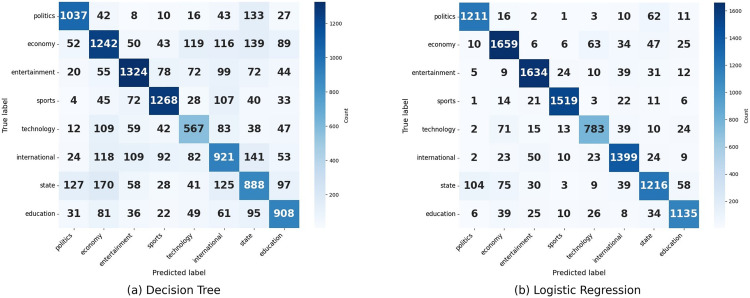
Confusion matrix of Decision Tree and Logistic Regression.

**Fig 10:**
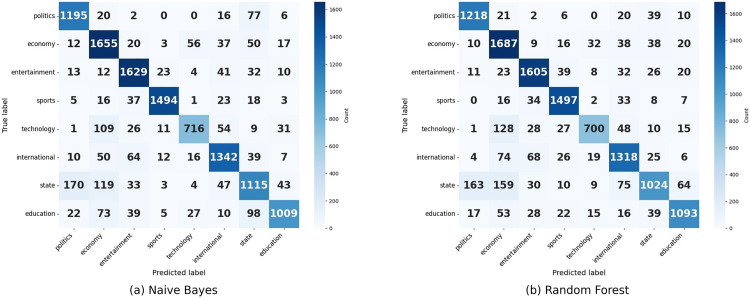
Confusion matrix of Naive Bayes and Random Forest.

**Fig 11:**
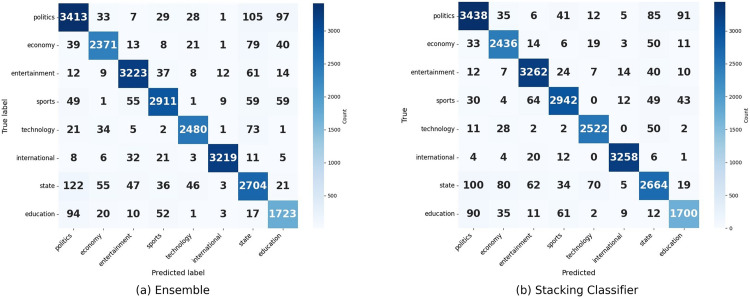
Confusion matrix of Ensemble and Stacking Classifier.

**Fig 12:**
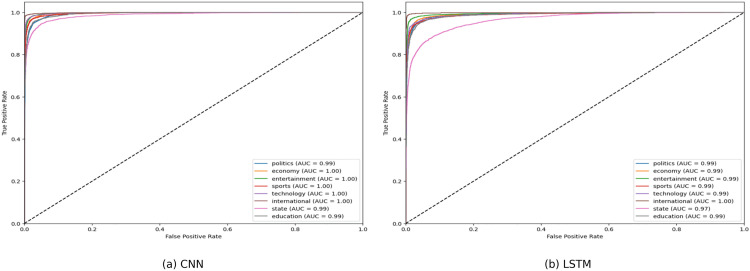
ROC curve of CNN and LSTM.

**Fig 13:**
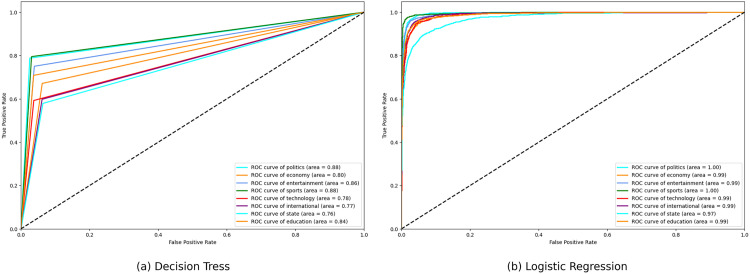
ROC curve of Decision Tress and Logistic Regression.

**Fig 14:**
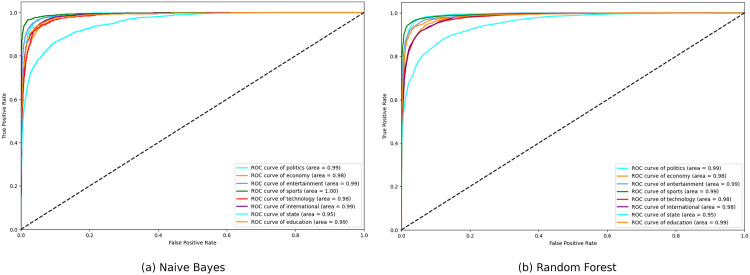
ROC curve of Naive Bayes and Random Forest.

**Fig 15:**
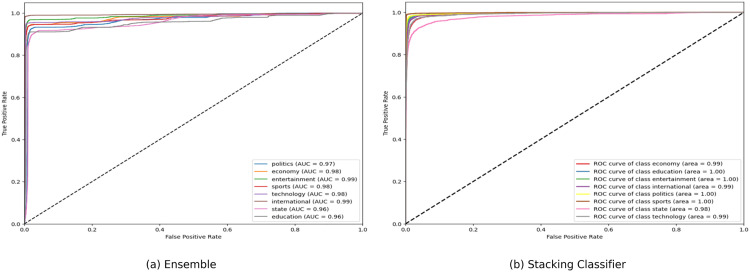
ROC curve of Ensemble and Stacking Classifier.

**Fig 16:**
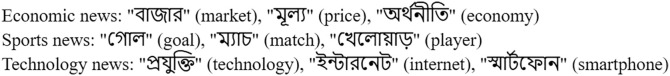
Some highlighted words form word-cloud.

The Funding statement for this article is incorrect. The correct Funding statement is as follows: This work was supported by King Saud University, Riyadh, Saudi Arabia, under ongoing Research Funding program (ORF-2025–951).
